# Juvenile arthritis disease activity score (JADAS) based on C-reactive protein predicts DMARD treatment in juvenile idiopathic arthritis in a Nordic multicenter cohort

**DOI:** 10.1186/1546-0096-10-S1-A42

**Published:** 2012-07-13

**Authors:** Ellen Nordal, Marek Zak, Lillemor Berntson, Kristiina Aalto, Suvi Peltonieli, Susan Nielsen, Troels Herlin, Bjørn Straume, Anders Fasth, Marite Rygg

**Affiliations:** 1 Århus University Hospital, Arhus, Denmark; 2 Norwegian University of Science and Technology, Trondheim, Norway; 3 Rigshospitalet Copenhagen, Copenhagen, Denmark; 4 University Hospital for Children and Adolescents, Helsinki, Finland; 5 University Hospital of North Norway, Tromso, Norway; 6 University of Gothenburg, Gothenburg, Sweden; 7 University of Helsinki, Helsinki, Finland; 8 University of Tromsø, Tromso, Norway; 9 Uppsala University Children's Hospital, Uppsala, Sweden

## Purpose

Juvenile Arthritis Disease Activity Score (JADAS) is a recently developed composite tool for scoring disease activity in juvenile idiopathic arthritis (JIA). JADAS consists of four items; the joint count, the physician and the patient’s/parent’s global assessment and the erythrocyte sedimentation rate (ESR) as an inflammatory marker. C-reactive protein (CRP) is often a more rapid and available test than ESR and has been suggested as an alternative inflammatory marker. The aim of the study was to validate and compare the CRP *versus* the ESR as an inflammatory marker in JADAS and to test whether higher JADAS scores were associated to DMARD treatment in a cohort of Nordic children with JIA in a near population-based setting.

## Methods

We included 514 consecutive cases of JIA from defined geographical areas of Denmark, Finland, Sweden and Norway with disease onset in 1997 to 2000. Clinical data and disease activity measures were registered according to a set protocol at regular follow-up visits from six to 147 months after onset. To calculate the JADAS, CRP was “normalized” to a value in the range 0-10 in a similar method as ESR, as described by Consolaro *et al* (1). Cut-off for CRP was <10mg/ml and for ESR <20mm/H. Spearman’s rank order correlation rho was used to evaluate the correlations between CRP and ESR, and the JADAS27 based on CRP and ESR. Logistic regression was used to test the first JADAS score available as a predictor of DMARD treatment, and whether mean JADAS score was associated to treatment with methotrexate and biologic agents in children with at least three JADAS scores available.

## Results

Of the 514 children 66% were girls, 51% had oligoarticular disease six months after onset and 3% were rheumatoid factor positive. The first visit when both CRP and ESR were taken was chosen for analysis. Correlation between corresponding CRP and ESR values from each visit was moderate (r= 0.57). There was a high correlation between JADAS27-CRP *versus* JADAS27-ESR (r=0.98). Bland-Altman plot of JADAS27-CRP *versus* JADAS27-ESR showed a high level of agreement (mean difference of 0.046 (CI -0.012 to 0.104)). Higher score of the first available JADAS27-CRP was significantly associated to treatment with methotrexate (OR= 1.12, 95%CI 1.07-1.17) and synthetic or biologic DMARDs (OR= 1.16, 95%CI 1.10-1.22) during the eight years follow-up after disease onset. Mean JADAS27-CRP also showed a significant association to treatment with methotrexate (OR= 1.37, 95%CI 1.19-1.57) and synthetic or biologic DMARDs (OR= 1.64, 95%CI 1.32-2.04) during the eight years follow-up after disease onset.

## Conclusion

JADAS based on CRP showed a high correlation with JADAS based on ESR in a multi-center Nordic cohort of juvenile idiopathic arthritis, indicating that CRP can be used as an alternative to ESR in the composite tool. High mean JADAS score during disease course was significantly associated to DMARD treatment in a prospective, longitudinal, multi-center Nordic JIA cohort. Further analyses on responsiveness to change of the JADAS-CRP are planned.

## Disclosure

Ellen Nordal: None; Marek Zak: None; Lillemor Berntson: None; Kristiina Aalto: None; Suvi Peltonieli: None; Susan Nielsen: None; Troels Herlin: None; Bjørn Straume: None; Anders Fasth: None; Marite Rygg: None.

